# FOXM1 regulates glycolysis in nasopharyngeal carcinoma cells through PDK1

**DOI:** 10.1111/jcmm.17413

**Published:** 2022-06-03

**Authors:** Qing Yang, Fang Wu, Yong Zhang, Rensheng Wang

**Affiliations:** ^1^ Department of Radiation Oncology The First Affiliated Hospital of Guangxi Medical University Nanning China

**Keywords:** FOXM1, glycolysis, nasopharyngeal carcinoma, PDK1, proliferation

## Abstract

The transcription factor forkhead box M1 (FOXM1) is a well‐known proto‐oncogene that plays a significant role in the pathogenesis of various human cancers. However, the regulatory role and underlying mechanisms of FOXM1 in nasopharyngeal carcinoma (NPC) metabolism remain unclear. We demonstrated that FOXM1 could positively regulate glycolysis in NPC cells. Functional studies have shown that pyruvate dehydrogenase kinase 1 (PDK1) is involved in FOXM1‐regulated lactate production, ATP generation and glycolysis. FOXM1 binds directly to the PDK1 promoter region and increases the expression of PDK1 at the transcriptional level, leading to the phosphorylation of pyruvate dehydrogenase (PDH) at serine 293, inhibiting its activity. Knocking down FOXM1 using specific short hairpin RNAs (shRNAs) can significantly decrease glycolysis and the expression of PDK1 in NPC cells. Furthermore, microenvironmental factors can increase the expression of FOXM1 by regulating hypoxia‐inducible factor 1α (HIF‐1α) expression. Clinical data and in vivo studies confirmed the positive roles of FOXM1/PDK1 in NPC proliferation and progression. In conclusion, our findings revealed that FOXM1 regulates glycolysis and proliferation of NPC through PDK1‐mediated PDH phosphorylation. Therefore, targeting the FOXM1‐PDK1 axis may be a potential therapeutic strategy for NPC.

## 1 INTRODUCTION

Nasopharyngeal carcinoma (NPC) is a common malignancy of the head and neck with a particularly high incidence in East and Southeast Asia.^1^ The prognosis of patients with NPC has dramatically improved due to advancements in radiotherapy and combined chemotherapy. However, approximately 30% of patients with NPC develop distant metastasis or recurrence.^2^ Therefore, a better understanding of the pathogenesis of NPC is essential for the development of novel therapeutics for it.

Recently, cancer metabolism has gained considerable attention in cancer research. As a recognized hallmark of cancer, aerobic glycolysis, also known as the Warburg effect, is a phenomenon wherein cancer cells undergo glycolysis instead of oxidative phosphorylation regardless of the presence of oxygen.^3^ Recent research has shown that altering metabolic gene expression in glycolysis is widely regarded as a desirable target for cancer therapeutics. For example, selectively targeting the glucose transporters GLUT1 and GLUT3 suppresses glycolysis, reduces the levels of glucose‐derived metabolites, and efficiently attenuates tumour cell growth.^4^ Hexokinase‐2 depletion inhibits glycolysis and markedly increases susceptibility to cell death and sorafenib treatment in hepatocellular carcinoma.^5^ The genetic depletion of lactate dehydrogenase A (LDHA) inhibits the proliferation of Ewing sarcoma cells, induces apoptosis, and reduces tumour growth.^6^ However, the selective blockade of these glycolysis‐related enzymes in cancer cells remains a critical challenge because it induces unwanted side effects, and these enzymes are ubiquitously expressed in all mammalian cells.^7^ Therefore, a better understanding of the exact mechanisms that promote the pathogenesis of nasopharyngeal carcinoma is urgently required.

Forkhead box M1 (FOXM1), a member of the Forkhead superfamily, is a transcription factor recognized as a master regulator of tumour development, cell cycle progression, invasion and metastasis in a variety of cancers, including hepatocellular carcinoma,^8^ lung adenocarcinoma,^9^ prostate cancer^10^ and ovarian cancer.^11^ Several studies have shown that FOXM1 overexpression was associated with aerobic glycolysis in some cancer cells.^12^, ^13^ Increasing evidence suggests that the overexpression of FOXM1 promotes NPC metastasis.^14^, ^15^, ^16^, ^17^ However, whether FOXM1 regulates glycolysis in NPC and its underlying mechanisms remain unclear.

In this study, we revealed that FOXM1 could positively regulate the glycolysis and proliferation of NPC cells by regulating pyruvate dehydrogenase kinase 1 (PDK1), one of the most crucial metabolic enzymes.

## 2 MATERIALS AND METHODS

### 2.1 Cell lines and culture conditions

A human nasal mucosal epithelial cell line (HNEpC) and NPC cell lines (CNE‐1, SUNE‐1, HONE‐1, and C666‐1) were all obtained from the Culture Collection of the Chinese Academy of Sciences. All cells were cultured in RPMI 1640 medium (Gibco) containing 10% foetal bovine serum (Biological Industries) and 1% penicillin–streptomycin liquid (Solarbio) at 37°C with 5% CO_2_ in a humidified incubator. Cultures with fewer than eight passages were used for all experiments. Hypoxic conditions were established by culturing cells at 37°C with 1% O_2_ and 5% CO_2_ in a modulator incubator. All cell lines were authenticated through short tandem repeat profiling and found to be free of mycoplasma contamination.

### 2.2 Treatment with hydrogen peroxide, N‐acetyl cysteine and transforming growth factor‐β1

Hydrogen peroxide (H_2_O_2_) (7722‐84‐1; Sigma) was used at concentrations of 0, 1, 5 and 10 μM; N‐acetyl cysteine (NAC) (HY‐B0215; MCE) was used at concentrations of 0, 1, 5 and 10 mM and transforming growth factor‐β1 (TGF‐β1) (100‐16A; PeproTech) was used at concentrations of 0, 1, 5 and 10 ng/ml.

### 2.3 Clinical samples

Nasopharyngitis tissues (*n* = 6) and NPC tissues (*n* = 12) were obtained from the First Affiliated Hospital of Guangxi Medical University. This study was approved by the Ethics Committee of the First Affiliated Hospital of Guangxi Medical University [approval no.2022‐KY‐E‐(068)]. All human samples were collected after receiving written informed consent from the patients, following the recognized ethical guidelines of the Belmont Report.

### 2.4 TCGA database search

Normalized gene‐level RNA‐seq and corresponding clinical data of normal mucosa samples (*n* = 44) and head and neck cancer (*n* = 502) from patients were selected from The Cancer Genome Atlas (TCGA) database (https://cancergenome.nih.gov/). Quantile normalization was performed to normalize mRNA expression (transcription fragments per million base pairs per thousand base fragments [FPKM]) data. The R statistical package (v.4.0.2) was used to analyse clinical and survival data. Gene set enrichment analysis (GSEA) was performed using GSEA software.

### 2.5 RNA extraction and qPCR

Total RNA was isolated from NPC cells and tissues using TRIzol reagent (Invitrogen), according to the manufacturer's protocol. cDNA was synthesized from the total mRNA using a high‐capacity cDNA reverse transcription kit (Takara) following the manufacturer's protocol. qPCR was then performed using a SYBR Green kit (Noblelab) with 0.2 μM primers on a Roche LightCycler 480 system under the following conditions: at 95°C for 2 min, followed by 45 cycles of amplification at 95°C for 10 s and 60°C for 20 s. Relative gene expression was normalized to β‐actin through quantification using the 2^−ΔΔ*C*t^ method. The primer sequences used for PCR are provided in Table S1.

### 2.6 Western blot analysis

Nasopharyngeal carcinoma cells were treated with radioimmunoprecipitation assay (RIPA) buffer containing 1% phenylmethylsulfonyl fluoride (PMSF), proteinase inhibitor and phosphatase inhibitor on ice to extract the total protein. The supernatant was collected via centrifugation at 12,000 g for 25 min at 4°C. Protein quantification was performed using a bicinchoninic acid (BCA) kit. Approximately 20–30 μg of protein was subjected to 8%–10% SDS‐PAGE and transferred onto PVDF membranes. The membranes were incubated with 5% non‐fat dried milk at room temperature for 1 h. Afterward, the membranes were incubated with primary antibodies at 4°C overnight and then with the corresponding secondary antibodies at room temperature for 1 h. Immunoreactive bands were scanned using an Amersham Imager 680 (GE Healthcare). The antibodies used are listed in Table S2.

### 2.7 Cell transfection

To overexpress FOXM1 and PDK1, full‐length FOXM1 and PDK1 cDNA sequences were cloned into PGMLV‐CMV‐MCS‐3 × Flag‐PGK‐Puro to generate overexpression plasmids (Genomeditech), which were then transfected into NPC cell lines using Lipofectamine 3000 (Invitrogen). An empty vector (EV) was used as a negative control. For FOXM1 knockdown, two designed shRNAs (Genomeditech) were transfected into NPC cells, according to the manufacturer's instructions. Non‐target shRNA (shCtrl) was used as a negative control. For PDK1 and HIF‐1α knockdown, synthesized duplex RNAi oligos (siRNA) targeting specific mRNA sequences (Genomeditech) were introduced into NPC cells using Lipofectamine 3000. A scrambled duplex RNA oligo (siCtrl) was used as the RNA control. Transfection efficiency was assessed via quantitative qPCR or western blotting. The shRNA‐ and siRNA‐targeting sequences are listed in Table S3.

### 2.8 Immunofluorescence staining

HONE‐1 and C666‐1 cells were seeded onto Falcon chamber slides. Once the cells reached 60% confluence, they were fixed with 4% paraformaldehyde and permeabilized with 0.5% Triton X‐100. The cells were then rinsed three times in cold phosphate‐buffered saline (PBS) and then incubated with anti‐PDK1 (1:500, ab202468; Abcam) primary antibody at 4°C overnight. Subsequently, the cells were incubated with Alexa Fluor® 594‐conjugated goat anti‐rabbit IgG‐H&L (1:500, ab150080; Abcam) at room temperature for 1 h. Finally, the slides were mounted using an antifade mounting medium with DAPI (Solarbio). Images of the cells were obtained using an inverted fluorescence microscope (Olympus Corporation).

### 2.9 Immunohistochemistry staining

Tumour tissues were fixed with 4% paraformaldehyde and cut into 4‐μm‐thick sections. Immunohistochemistry (IHC) was performed according to the protocol. The sections were incubated with rabbit anti‐FOXM1 (1:100, 20,459, CST), anti‐PDK1 (1:100, 18,262‐1‐AP; Proteintech), anti‐GLUT1 (1:200, 66,290‐1‐Ig; Proteintech), anti‐ LDHA (1:200, 66,287‐1‐Ig; Proteintech) and anti‐HK2 (1:100, 22,029‐1‐AP; Proteintech) antibodies overnight at 4°C. The sections were then sequentially incubated with a biotinylated secondary antibody (PV‐9000; ZSGB‐Bio) to detect the primary antibodies. Images were obtained using TissueFAXS systems (TissueGnostics).

### 2.10 Glucose uptake assays

Indicated HONE‐1 and C666‐1 cells were incubated in a glucose‐free medium at 37°C with 5% CO_2_ for 1 h, incubated in a glucose‐free medium with 50 μM 2‐NBDG (HY‐116215; MCE) for 30 min, and then the cells were washed with PBS thrice. The fluorescence intensity of the cells was measured using a flow cytometry system (BD Biosciences).

### 2.11 Measurement of ATP generation

Cellular ATP levels were determined using an ATP assay kit (S0026; Beyotime) according to the manufacturer's instructions. Luminescence was measured using a luminescence reader, and the values were normalized to the amount of protein per sample.

### 2.12 Measurement of lactate production

Cells were seeded in 6‐well plates and cultured for 24 h. The lactate concentration in the culture medium was measured using a lactate assay kit (Nanjing Jiancheng Corporation). Lactate production was normalized to the number of cells.

### 2.13 Measurement of extracellular acidification rate

Cells were seeded in Agilent Seahorse XFe96 plates at a density of 5 × 10^4^ cells/well and allowed to adhere for 8 h in a standard incubator. Cells were then equilibrated with XF base media at 37°C for 1 h in an incubator without CO_2_ and then serum‐starved for 1 h in glucose‐free media‐containing treatments. Extracellular acidification rate (ECAR) was then measured using a glycolytic stress test kit (103020–100, Agilent Technologies). Briefly, the cells were treated with the sequential addition of glucose (10 mM), oligomycin (1.0 μM), and 2‐DG (50 nM), as described in the protocol of the XF glycolysis stress test using a Seahorse XFe96 Extracellular Flux Analyser (Agilent Technologies).

### 2.14 EdU proliferation assay

An EdU (5‐ethynyl‐2‐deoxyuridine) assay kit (RiboBio) was used to measure cell proliferation, as described previously.^18^

### 2.15 Cell counting kit 8 assay

Cells (5 × 10^3^) transfected with different nucleic acids were seeded in 96‐well plates and cultured for 1, 2, 3 or 4 days. Subsequently, Cell counting kit 8 (CCK‐8) solution (GK10001; Glpbio) was added to the cells, and incubation was continued for another 1 h. The optical density (OD) was measured at 450 nm using a microplate spectrophotometer (Thermo Fisher Scientific).

### 2.16 Luciferase reporter assay

Wild‐type or mutant PDK1 promoters were cloned into a pGL3‐basic firefly luciferase reporter plasmid to generate pGL3‐PDK1‐WT‐Luc, pGL3‐PDK1‐Mut1‐Luc and pGL3‐PDK1‐Mut2‐Luc (Genomeditech) plasmids. Plasmids were co‐transfected with pRL‐TK into shCtrl‐ or shFOXM1‐treated HONE‐1 cells for 48 h. Firefly luciferase (F‐Luc) and Renilla luciferase (R‐Luc) activity were assayed using a Dual‐Glo Luciferase Assay System (Promega) according to the manufacturer's instructions. The activity of pRL‐TK encoding Renilla luciferase (R‐Luc) was used to normalize firefly luciferase (F‐Luc) activity.

### 2.17 Chromatin immunoprecipitation assay

Cells were harvested for chromatin immunoprecipitation (ChIP) using a ChIP Kit (Millipore), following the manufacturer's protocol. Briefly, the chromatin was extracted and incubated with IgG and anti‐FOXM1 (1:100; 20,459; CST) antibody overnight at 4°C. Antibody/chromatin complexes were precipitated using protein G agarose. The antibody/chromatin/protein G agarose complexes were resuspended and centrifuged to collect protein/DNA complexes. The protein/DNA crosslinks were cleaved to yield free DNA. The obtained DNA was purified, and qPCR was performed to quantify FOXM1 binding to the PDK1 promoter fragments. Relative enrichment was normalized to the negative‐antibody control (IgG) using the 2^−ΔΔ*C*t^ quantification method. The sequences of the primers used to detect the promoter regions for FOXM1 binding are listed in Table S1.

### 2.18 Xenograft mouse model

Female BALB/c nude mice (4 weeks old, five mice per group) were purchased from Vital River Laboratories and subjected to tumour implantation. HONE‐1 cells expressing shCtrl and shFOXM1 were transfected with an empty vector or PDK1 overexpression plasmid, and the resulting cells were subcutaneously injected into the dorsal flanks of the mice (1 × 10^6^ cells/mouse). Tumour growth was monitored weekly after transplantation using callipers. Four weeks after transplantation, the mice were euthanized to harvest the tumours, and the tumour volume and mass were measured. Tumours obtained from the mice were examined using immunohistochemistry and western blotting. All experimental procedures were approved by the ethics committee of the First Affiliated Hospital of Guangxi Medical University [approval no.2022‐KY‐E‐(068)].

### 2.19 Statistical analysis

Data are reported as mean ± SD from at least three independent experiments. Two‐tailed unpaired Student's *t*‐test between two groups and one‐way ANOVA were used for multiple comparisons. The Wilcoxon signed‐rank test was used to determine the difference in FOXM1 and PDK1 expression between normal mucosa and head and neck cancer from the TCGA database. Pearson's correlation test was used to analyse the correlation between FOXM1 and PDK1 expression. Survival analyses were performed using the Kaplan–Meier method and assessed using the log‐rank test. Differences in means were considered statistically significant at *p* < 0.05. ns, not significant; *p < 0.05; **p < 0.01; ***p < 0.001.

## 3 RESULTS

### 3.1 FOXM1 is highly expressed in NPC and predicts a worse prognosis

**FIGURE 1:**
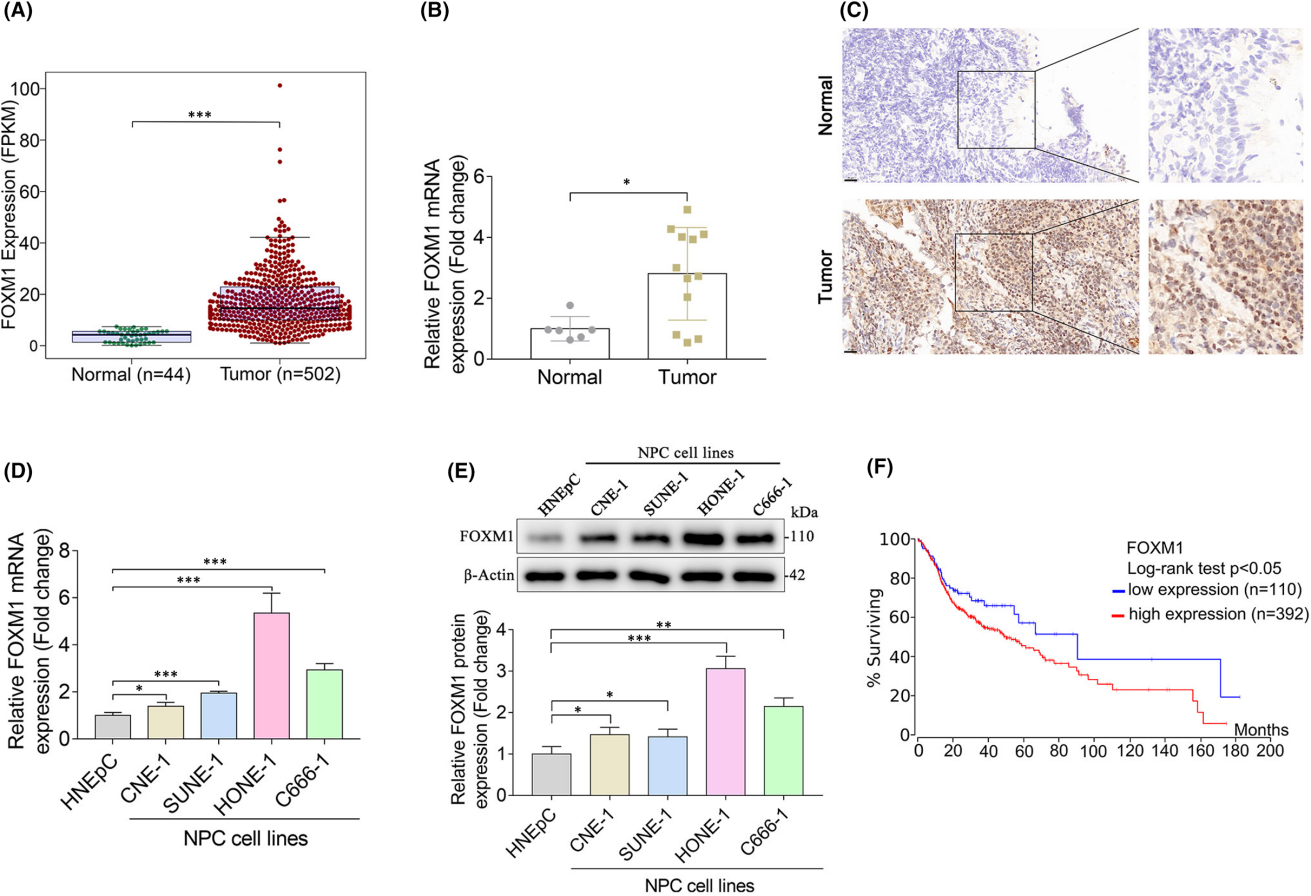
FOXM1 is highly expressed in nasopharyngeal carcinoma (NPC) and predicts a worse prognosis. (A) FOXM1 expression levels in non‐cancerous (*n* = 44) and head and neck cancer (*n* = 502) samples in the TCGA cohort. (B) The mRNA expression of FOXM1 in non‐cancerous nasopharyngeal samples (*n* = 6) and NPC tissue (*n* = 12) detected via qPCR. (C) IHC analysis of FOXM1 protein expression in non‐cancerous nasopharyngeal and NPC tissue. Scale bars, 20 μm. (D–E) FOXM1 expression levels in HNEpC and human NPC cell lines were detected via qPCR (D) and western blot (E). HNEpC is an immortalized normal nasopharyngeal epithelial cell line, while CNE‐1, SUNE‐1, HONE‐1 and C666‐1 are human NPC cell lines. (F) The prognostic value of FOXM1 expression in head and neck cancer (*n* = 502) was analysed using data from the TCGA database. *p < 0.05; ***p < 0.001

Previous studies have documented that FOXM1 is aberrantly expressed in human malignancies.^12^, ^19^ In this study, we analysed the expression of FOXM1 in a cohort of patients from TCGA with normal mucosa and head and neck cancer. We observed that FOXM1 expression was higher in head and neck cancer samples than in the normal mucosa (Figure 1A). To validate the TCGA results, we examined the expression status of FOXM1 using quantitative real‐time PCR (qPCR) in non‐cancerous nasopharyngeal samples and NPC tissue. We found that FOXM1 expression was significantly higher in NPC tissues than in normal nasal mucosa (Figure 1B,C). In addition, FOXM1 expression was dramatically increased in NPC cell lines, particularly in HONE‐1 and C666‐1 cells (Figure 1D,E). Analysis of the survival curves from data obtained in the TCGA database indicated that patients with head and neck cancer with lower FOXM1 expression had significantly longer survival than those with higher FOXM1 expression (Figure 1F). These data suggest that FOXM1 is involved in NPC progression, and its expression is correlated with patient survival.

### 3.2 FOXM1 promotes glycolysis and the proliferation of NPC cells

**FIGURE 2:**
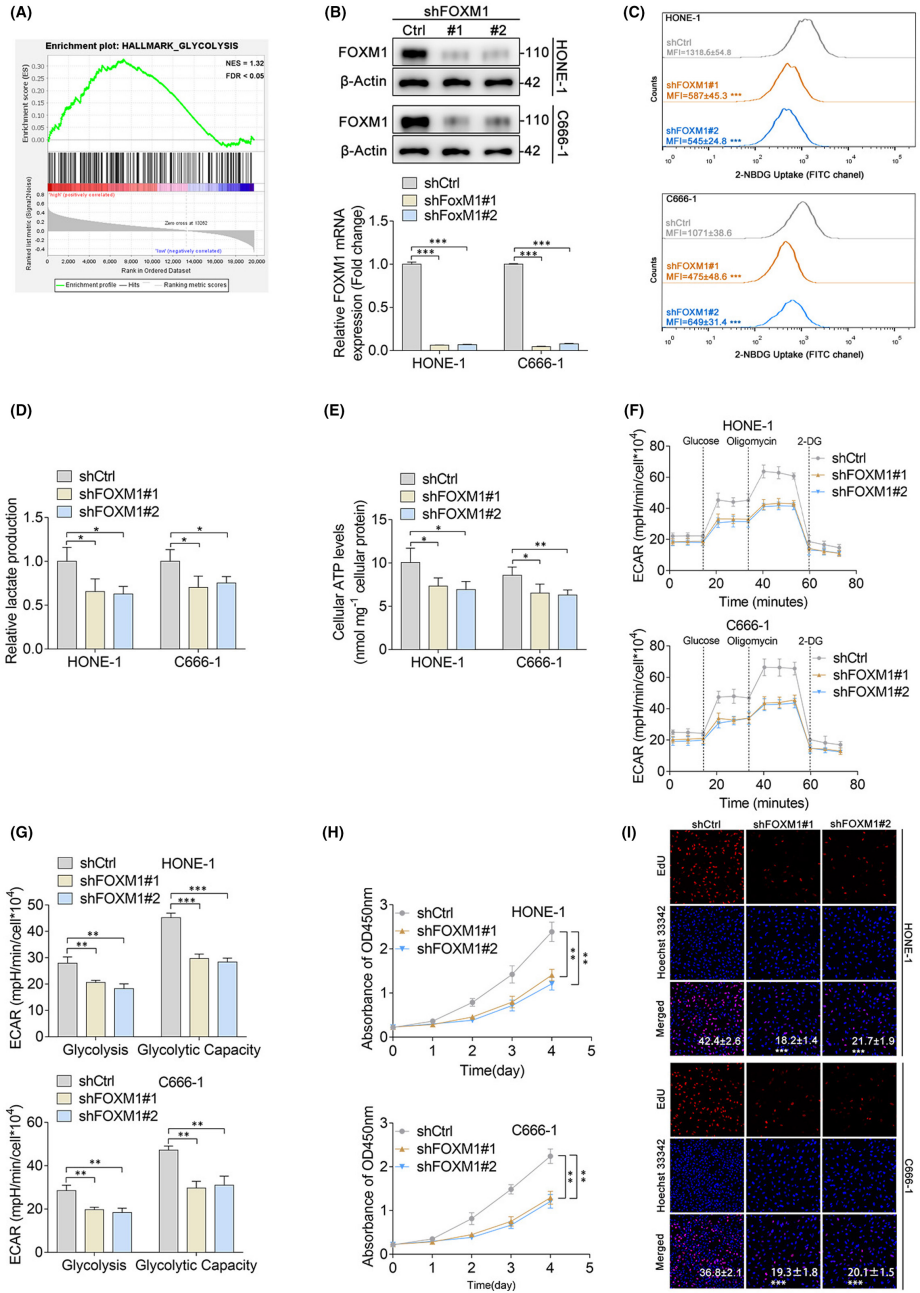
FOXM1 promotes glycolysis and the proliferation of nasopharyngeal carcinoma (NPC) cells. (A) Pathway enrichment analysis suggested that FOXM1 was enriched in glycolysis. Pathway analysis was performed using the GSEA method, which was based on an empirical permutation test procedure. (B) Western blot and qPCR results show the transfection efficiency of FOXM1 knockdown in HONE‐1 and C666‐1 cells. (C) Intracellular glucose uptake in shCtrl‐ and shFOXM1‐treated HONE‐1 and C666‐1 cells was quantified via 2‐NBDG staining. (D–E) Lactate production (D) and intracellular ATP levels (E) of shCtrl‐ and shFOXM1‐treated HONE‐1 and C666‐1 cells. (F) The ECAR of shCtrl‐ and shFOXM1‐treated HONE‐1 and C666‐1 cells was measured using a Seahorse XFe96 Extracellular Flux analyser. (G) Statistical analysis of the effects of FOXM1 knockdown on glycolysis and glycolytic capacity. (H–I) The proliferation of shCtrl‐ and shFOXM1‐treated HONE‐1 and C666‐1 cells were determined using a CCK‐8 (H) and EdU proliferation assay (I). *p < 0.05; **p < 0.01; ***p < 0.001

It is generally perceived that cancer cells shift their glucose metabolism pattern to aerobic glycolysis for a growth advantage. Based on the results that FOXM1 was aberrantly expressed in NPC samples and NPC cell lines, and that the expression of FOXM1 could predict the prognosis of head and neck cancer, we hypothesized that FOXM1 participates in the regulation of aerobic glycolysis. Pathway analysis indicated that FOXM1 expression positively correlated with glycolysis (Figure 2A). To further test our hypothesis, we silenced FOXM1 expression using short hairpin RNAs (shRNAs) in HONE‐1 and C666‐1 NPC cell lines, which have a relatively high endogenous expression of FOXM1, and transfection efficiency was examined via western blotting and qPCR (Figure 1B). Cells with FOXM1 knockdown exhibited significantly lower glucose uptake as evidenced by decreased 2‐NBDG (a fluorescent indicator for direct glucose uptake measurement) fluorescence (Figure 2C), lactate production (Figure 2D), and intracellular ATP levels (Figure 2E) compared to control cells. To functionally characterize the FOXM1‐induced metabolic phenotype, negative control or FOXM1‐knockdown NPC cells were subjected to a glycolysis stress test. The glycolysis stress test measures the extracellular acidification rate (ECAR) after adding glucose, oligomycin A, and 2‐DG using an extracellular flux analyser. The results showed that the depletion of FOXM1 strikingly reduced both basal glycolysis and glycolytic capacity in HONE‐1 and C666‐1 cells (Figure 2F,G). As high levels of glycolytic intermediates play a fundamental role in supporting cell growth^20^ and FOXM1 is involved in NPC cell aerobic glycolysis, we further investigated the functional role of FOXM1 in cellular behaviour. FOXM1 knockdown in HONE‐1 and C666‐1 cells strikingly inhibited cell proliferation, as shown by the results of the CCK‐8 assay (Figure 2H). In agreement with these findings, FOXM1 silencing significantly decreased the percentage of EdU‐positive cells compared to the control group for HONE‐1 and C666‐1 cells (Figure 2I). Taken together, these data suggest that FOXM1 positively regulates glycolysis and the proliferation of NPC cells.

### 3.3 FOXM1 transcriptionally regulates PDK1, inhibiting mitochondrial PDH activity

**FIGURE 3:**
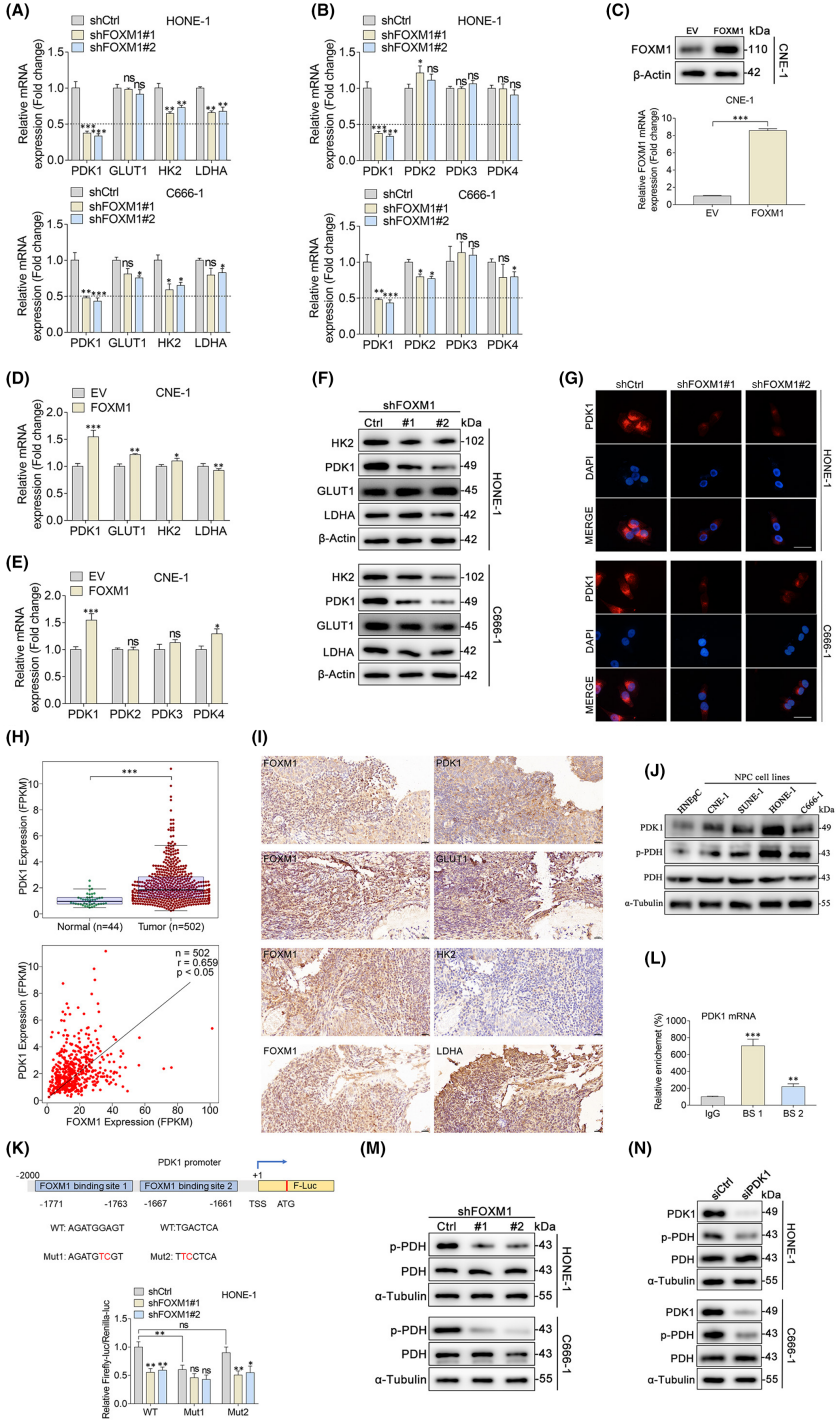
FOXM1 transcriptionally regulates the expression of PDK1. (A) qPCR detection of the mRNA expression of PDK1, GLUT1, HK2 and LDHA in HONE‐1 and C666‐1 cells after knocking down FOXM1 expression. (B) qPCR detection of the mRNA expression of PDK1, PDK2, PDK3 and PDK4 in HONE‐1 and C666‐1 cells after knocking down FOXM1 expression. (C) Western blot and qPCR data show the transfection efficiency of FOXM1 overexpression in CNE‐1 cells. (D) CNE‐1 cells were transfected with empty vector control or FOXM1 construct for 48 h, and the mRNA expression of PDK1, GLUT1, HK2 and LDHA was measured via qPCR. (E) CNE‐1 cells were transfected with empty vector control or FOXM1 construct for 48 h, and the mRNA expression of PDK1, PDK2, PDK3 and PDK4 was measured via qPCR. (F) The protein expression of PDK1, GLUT1, HK2 and LDHA in HONE‐1 and C666‐1 cells after FOXM1 knockdown was measured via western blot. (G) Immunofluorescence staining of PDK1 expression in HONE‐1 and C666‐1 cells after knocking down FOXM1 expression. Scale bar, 50 μm. (H) PDK1 expression levels in non‐cancerous samples (*n* = 44) and head and neck cancer (*n* = 502) (above), and the correlation between FOXM1 and PDK1 expression in head and neck cancer (*n* = 502) from the TCGA database (below). (I) Representative immunohistochemical images show FOXM1, PDK1, GLUT1, HK2 and LDHA in the serial sections of nasopharyngeal carcinoma (NPC) tissue. Scale bars, 20 μm. (J) PDK1 and p‐PDH expression levels in HNEpC and human NPC cell lines were detected via western blot. (K) The potential binding sites and mutant sites of FOXM1 binding to the PDK1 promoter. HONE‐1 cells were co‐transfected with pGL3‐PDK1‐WT‐Luc, pGL3‐PDK1‐Mut1‐Luc, pGL3‐PDK1‐Mut2‐Luc, pRL‐TK plasmid and shCtrl or shFOXM1 for 48 h. The results are presented as the ratio between the activity of the reporter plasmid and that of pRL‐TK. (L) ChIP‐PCR verified the binding between FOXM1 and the promoter of PDK1 at potential binding sites 1 and 2. Immunoglobulin G (IgG) was used as the negative control. (M) The protein expression of p‐PDH (Ser293) and PDH in HONE‐1 and C666‐1 cells after FOXM1 knockdown was measured via western blot. (N) HONE‐1 and C666‐1 cells were transfected with negative control siRNA (siCtrl) or siRNA for PDK1 (siPDK1) for 48 h, and western blot was performed to detect the protein levels of p‐PDH (Ser293) and PDH. ns, not significant; *p < 0.05; **p < 0.01; ***p < 0.001

Recent studies indicate that many key oncogenic signalling pathways regulate cancer metabolism by regulating glycolytic enzyme expression.^21^, ^22^ To further investigate the mechanism by which FOXM1 regulates glycolysis, we examined the effects of FOXM1 on the expression of glycolytic enzymes. Interestingly, the downregulation of FOXM1 reduced PDK1 mRNA levels, whereas the most important glycolytic enzymes, including GLUT1, HK2 and LDHA, showed no obvious changes in mRNA levels in HONE‐1 and C666‐1 cells (Figure 3A). We then sought to confirm the effect of FOXM1 knockdown on the mRNA expression of other PDK enzymes. However, the results displayed no obvious reduction in the levels of these enzymes (Figure 3B). In addition, the FOXM1‐overexpressing and control NPC cell lines were obtained via plasmid transfection of the CNE‐1 cell line, which had a relatively low endogenous expression of FOXM1 (Figure 3C). As expected, FOXM1 overexpression dramatically increased PDK1 mRNA levels, whereas the elevation of other glycolytic enzymes was not significant in CNE‐1 cells (Figure 3D,E). Similarly, FOXM1 knockdown significantly decreased the protein expression of PDK1, but the differences did not reach statistical significance in GLUT1, HK2 and LDHA (Figure 3F–G). We further found that PDK1 expression was also markedly elevated in head and neck cancer, and the expression of PDK1 was significantly and positively correlated with the expression of FOXM1 in patients with head and neck cancer from the TCGA database (Figure 3H). Besides, a positive association of high expression of FOXM1 in NPC tissues with PDK1 (Figure 3I). Moreover, PDK1 expression was increased in NPC cell lines (Figure 3J). These data indicate that FOXM1 positively regulates the expression of PDK1 in NPC cells.

FOXM1 binds to a promoter to regulate gene expression as a transcription factor. Therefore, we speculated that FOXM1 could bind to the PDK1 promoter and regulate its expression. To validate our hypothesis, we identified two putative FOXM1‐binding sites within the PDK1 promoter regions (BS1 and BS2) located upstream of the transcription start site (TSS) of PDK1 (Figure 3K). Subsequently, the wild‐type or mutant PDK1 promoter was cloned into the pGL3‐basic firefly luciferase reporter plasmid to generate pGL3‐PDK1‐WT‐Luc, pGL3‐PDK1‐Mut1‐Luc and pGL3‐PDK1‐Mut2‐Luc plasmids, and luciferase assays were performed. The results demonstrated that the knockdown of FOXM1 resulted in a lower level of luciferase activity in pGL3‐PDK1‐WT and pGL3‐PDK1‐Mut2‐Luc. In contrast, the inhibitory effect of FOXM1 knockdown was attenuated in pGL3‐PDK1‐Mut1‐Luc, indicating that FOXM1 positively regulates PDK1 transcriptional activity at binding site 1 (Figure 3L). In parallel, we carried out chromatin immunoprecipitation (ChIP)‐qPCR analysis to evaluate if FOXM1 binds to the PDK1 promoter. Consistent with the luciferase reporter assays, ChIP‐qPCR showed that the binding of FOXM1 to potential binding site 1 was much greater than that of binding site 2 in HONE‐1 cells (Figure 3K). Taken together, these findings suggest that FOXM1 regulates PDK1 expression by interacting with FOXM1‐binding sites in the PDK1 promoter region.

High PDK1 expression leads to enhanced phosphorylation of the pyruvate dehydrogenase complex and reduced PDH activity.^23^ Western blot analysis clearly showed that pyruvate dehydrogenase E1‐α phosphorylation at Ser293, the most frequent and efficient phosphorylation site to sufficiently inhibit PDH activity, was notably reduced after FOXM1 knockdown (Figure 3M). Interestingly, FOXM1 knockdown did not lead to changes in the expression of pyruvate dehydrogenase in either HONE‐1 or C666‐1 NPC cells (Figure 3M), strongly supporting that diminished pyruvate dehydrogenase E1‐α phosphorylation was driven by the previously observed effect of FOXM1 knockdown on PDK1 expression. To further test the putative role of PDK1 in inhibiting PDH activity, we measured the phosphorylation of pyruvate dehydrogenase, which inhibits PDK1 expression via siRNA. We detected a rapid and visually discernible de‐phosphorylation of Ser293 in HONE‐1 and C666‐1 cells after PDK1 knockdown (Figure 3N). Taken together, these findings indicate that PDK1 is a direct transcriptional target of FOXM1 that inhibits mitochondrial PDH activity.

### 3.4 PDK1 is involved in FOXM1‐regulated glycolysis and cell proliferation

**FIGURE 4:**
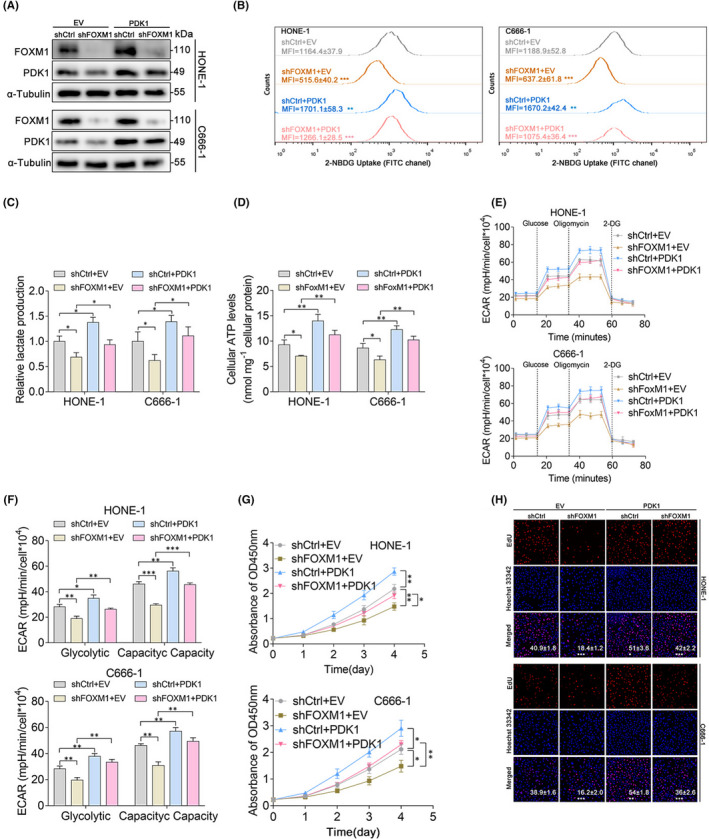
PDK1 is involved in FOXM1‐regulated glycolysis and cell proliferation. (A) The expression of PDK1 in shCtrl‐ and shFOXM1‐treated HONE‐1 and C666‐1 cells stably transfected with empty vector (EV) or PDK1 constructs was checked via western blot analysis. (B) Intracellular glucose uptake of shCtrl‐ and shFOXM1‐treated HONE‐1 and C666‐1 cells transfected with empty vector or PDK1 constructs via staining with 2‐NBDG. (C–D) Lactate production (C) and intracellular ATP levels (D) of shCtrl‐ and shFOXM1‐treated HONE‐1 and C666‐1 cells transfected with empty vector or PDK1 constructs. (E) ECAR measurement of shCtrl‐ and shFOXM1‐treated HONE‐1 and C666‐1 cells transfected with empty vector or PDK1 constructs using a Seahorse XFe96 Extracellular Flux analyser. (F) Statistical analysis of the effects of FOXM1 knockdown or PDK1 overexpression on glycolysis and glycolytic capacity. (G–H) The proliferation of shCtrl‐ and shFOXM1‐treated HONE‐1 and C666‐1 cells transfected with empty vector or PDK1 constructs were checked via the CCK‐8 assay (G) and EdU proliferation assay (H). *p < 0.05; **p < 0.01; ***p < 0.001

Since PDK1 is a critical enzyme that regulates glycolytic metabolism in cancer cells and our results have demonstrated that PDK1 is regulated by FOXM1, we next sought to determine whether PDK1 is involved in the elevated glycolysis and cell proliferation rates regulated by FOXM1. To investigate this, we overexpressed PDK1 in HONE‐1 and C666‐1 cells expressing shCtrl or shFOXM1 (Figure 4A). Overexpression of PDK1 reversed the suppressed glucose uptake of FOXM1‐knockdown HONE‐1 and C666‐1 cells compared to their corresponding control cells (Figure 4B). Consistently, PDK1 also attenuated the shFOXM1‐mediated decrease in lactate production rate (Figure 4C) and intracellular ATP levels (Figure 4D) in HONE‐1 and C666‐1 cells. Furthermore, the forced expression of PDK1 rescued the impairment in glycolysis and glycolytic capacity in HONE‐1 and C666‐1 cells (Figure 4E–F). These results suggested that PDK1 is involved in FOXM1‐regulated glycolysis in NPC cells. Furthermore, our results also indicated that PDK1 predominantly attenuated the shFOXM1‐mediated suppression of HONE‐1 and C666‐1 cell proliferation (Figure 4G–H). Collectively, these data strongly suggest that the enhancement of glycolysis and proliferation by FOXM1 depends on PDK1 in NPC cells.

### 3.5 Microenvironment‐mediated HIF‐1α stabilization enhances the expression of FOXM1

**FIGURE 5:**
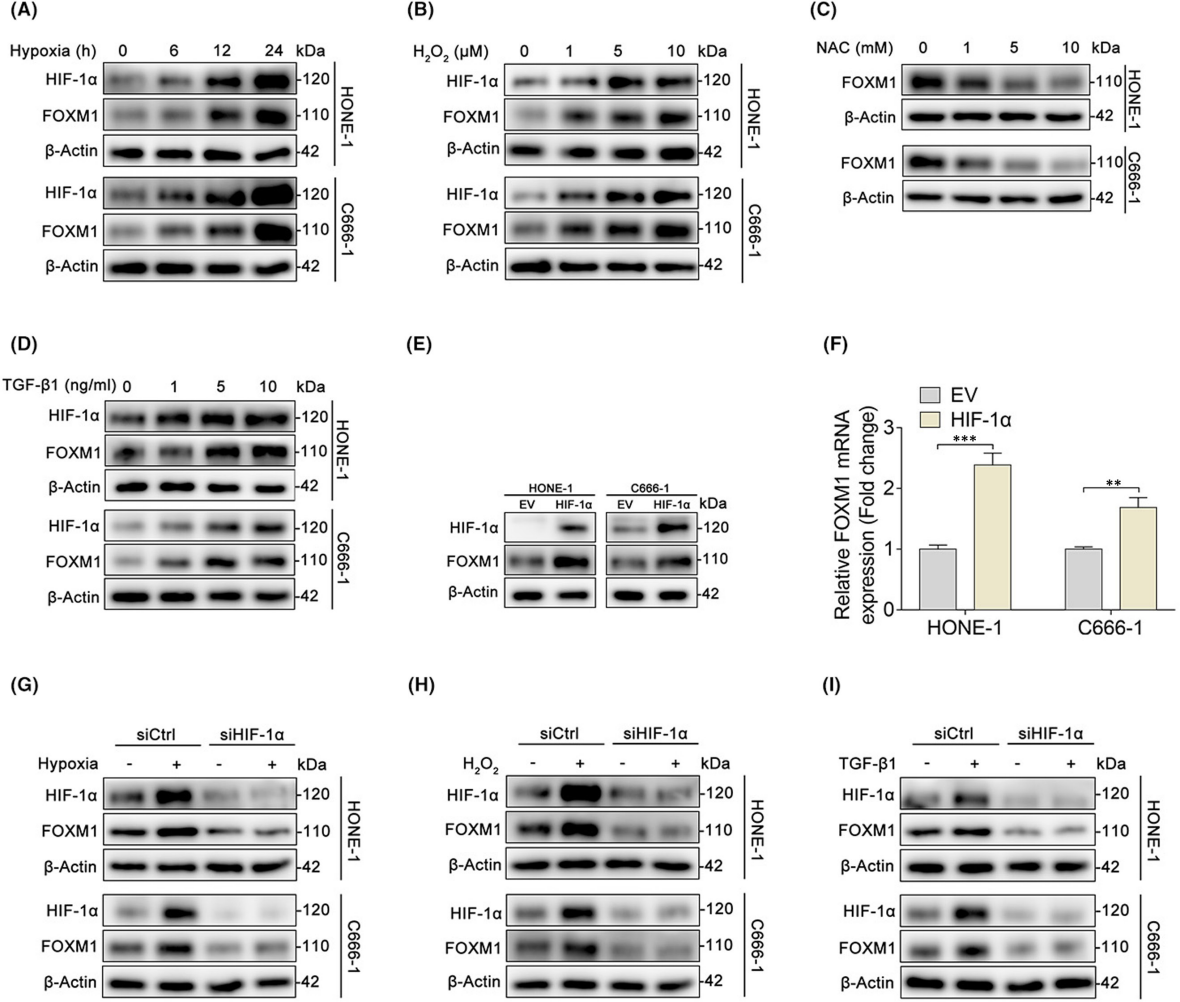
Microenvironment‐mediated HIF‐1α stabilization enhances the expression of FOXM1. (A) Western blot detection of HIF‐1α and FOXM1 expression in HONE‐1 and C666‐1 cells exposed to hypoxia (1% O_2_) or normoxia for 0, 6, 12 or 24 h. (B) Western blot detection of HIF‐1α and FOXM1 expression in HONE‐1 and C666‐1 cells exposed to H_2_O_2_ at concentrations of 0, 1, 5 or 10 μM for 24 h. (C) Western blot detection of FOXM1 expression in HONE‐1 and C666‐1 cells exposed to NAC at concentrations of 0, 1, 5 or 10 mM for 24 h. (D) Western blot detection of HIF‐1α and FOXM1 expression in HONE‐1 and C666‐1 cells exposed to cytokines (10 ng/ml TGF‐β1 to simulate the tumour microenvironment) for 0, 6, 12 or 24 h. (E–F) HONE‐1 and C666‐1 cells were transfected with empty vector or HIF‐1α constructs for 48 h, and the expression of HIF‐1α and FOXM1 were checked via western blot (E) and qPCR analysis (F). (G–I) HONE‐1 and C666‐1 cells were transfected with negative control siRNA (siCtrl) or siRNA for HIF‐1α (siHIF‐1α) and treated with hypoxia (1% O_2_) (G), H_2_O_2_ (10 μM) (H), or TGF‐β1 (10 ng/ml) (I) for 24 h. Western blot was then performed to detect the protein levels of HIF‐1α and FOXM1. **p < 0.01; ***p < 0.001

Multiple microenvironmental factors are critical for promoting cancer progression; therefore, we investigated whether these factors affect FOXM1 expression status in NPC cells. Intratumoral hypoxia is a common condition in human cancer that leads to increased activity of hypoxia‐inducible factors (HIFs), which regulate the expression of genes that contribute to cancer progression.^24^ Therefore, we extended our analysis to hypoxic conditions by detecting FOXM1 expression in cells exposed to severe hypoxia or normoxia over a gradient time course. Our data showed that HIF‐1α and FOXM1 levels were markedly increased after exposure to hypoxic conditions for 24 h (Figure 5A). As elevated reactive oxygen species (ROS) levels are implicated in the promotion of cancer cell growth and the metastatic progression,^25^ we examined whether ROS was critical for the expression of FOXM1. Treatment with hydrogen peroxide (H_2_O_2_) significantly increased the expression of FOXM1 (Figure 5B). In contrast, a decrease in FOXM1 protein levels was observed after treatment with N‐acetylcysteine (NAC), a well‐known antioxidant (Figure 5C). Accumulating evidence indicates that cytokines synthesized and released from the tumour microenvironment promote tumour growth and invasion.^26^ To validate the relationship between cytokine levels and FOXM1 expression, we treated cells with recombinant human TGF‐β1, a well‐known cytokine predominantly released from the tumour microenvironment. TGF‐β1 treatment also led to FOXM1 upregulation (Figure 5D). Increased expression of HIF‐1α has been shown to directly activate target genes^24^ and our results indicate that HIF‐1α was upregulated when exposed to microenvironmental factors (Figure 5A–C). To explore whether HIF‐1α directly interacts with FOXM1, NPC cells were transfected with an expression plasmid of HIF‐1α. Consistently, HIF‐1α overexpression significantly upregulated FOXM1 protein (Figure 5E) and mRNA levels (Figure 5F). To elucidate the underlying role of HIF‐1α in regulating microenvironment‐induced FOXM1 expression, we knocked down HIF‐1α expression in HONE‐1 and C666‐1 cells using a specific siRNA. Western blot analysis showed that HIF‐1α depletion significantly inhibited hypoxia‐ (Figure 5G), ROS‐ (Figure 5H) and TGF‐β1‐ (Figure 5I) induced FOXM1 protein expression. Altogether, these findings indicate that the tumour microenvironment‐induced stabilization of HIF‐1α is involved in the regulation of FOXM1 expression status.

### 3.6 PDK1 is involved in FOXM1‐regulated cancer progression in vivo

**FIGURE 6:**
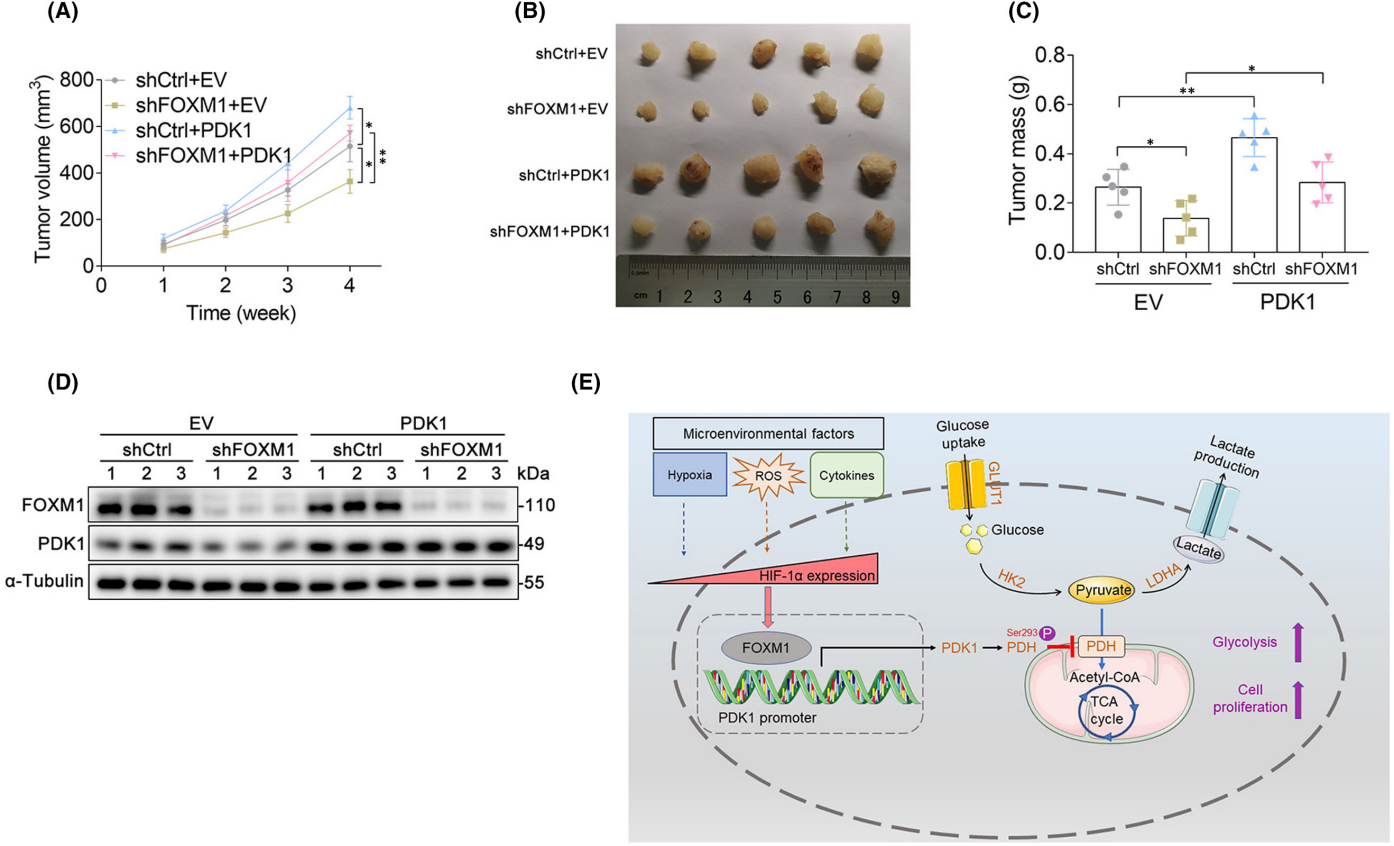
PDK1 is involved in FOXM1‐mediated cancer progression in vivo. (A) The tumour growth curves of shCtrl‐ and shFOXM1‐treated HONE‐1 cells stably transfected with empty vector or PDK1 constructs after being injected into nude mice. Tumour sizes were measured every week using a calliper. (B–C) Tumours were extracted, and the tumour sizes (B) and tumour masses (C) were measured at the end of the experiment. (D) Western blot was performed to detect the protein levels of FOXM1 and PDK1 in the tumour tissues of mice. (E) Graphical abstract showing that microenvironmental factors induce FOXM1, which transcriptionally activates PDK1 by directly binding to the promoter of PDK1. This interaction leads to PDH phosphorylation at Ser293 and promotes glycolysis and proliferation in nasopharyngeal carcinoma (NPC). *p < 0.05; **p < 0.01

It is widely accepted that glycolysis and ATP generation are critical for cancer progression, including cancer cell proliferation, growth, and metastasis.^20^ To further determine the function of FOXM1‐PDK1 signalling in NPC, we extended our investigation to in vivo experiments. Consistently, the xenograft model confirmed that the forced expression of PDK1 attenuated the suppressive effect of shFOXM1‐treated HONE‐1 cells on the in vivo tumour growth rate (Figure 6A), tumour size (Figure 6B), and tumour mass (Figure 6C). Western blotting of tumour tissue lysates revealed that PDK1 protein levels were significantly decreased after the knockdown of FOXM1, while the protein levels of PDK1 were restored by upregulating PDK1 expression (Figure 6D), suggesting that the knockdown of FOXM1 can regulate the expression of PDK1 in vivo. Of note, shFOXM1 was also observed to affect the growth of PDK1‐overexpressing cells in vivo (Figure 6A–C), possibly by inhibiting endogenous PDK1 expression or through other unknown mechanisms. Taken together, these results suggest that PDK1 is involved in FOXM1‐mediated tumour growth in xenograft mice.

## 4 DISCUSSION

Here, we report that the transcription factor FOXM1 activates PDK1, a glycolysis gatekeeper, and promotes glycolysis, leading to cancer progression and poor prognosis in NPC. Our results indicate for the first time that FOXM1 binds directly to the PDK1 promoter region and transcriptionally activates PDK1, leading to PDH phosphorylation and the regulation of glycolysis. Importantly, our data further demonstrated that PDK1 expression was critical for FOXM1‐mediated cancer growth in vitro and in vivo. Thus, these findings provide oncogenic and mechanistic evidence regarding the involvement of FOXM1 in glycolysis and cancer progression and suggest a novel therapeutic target for NPC (Figure 6E).

The oncogenic transcription factor FOXM1 is markedly overexpressed in many types of cancer cells, including pancreatic cancer,^12^ lung adenocarcinoma^9^ and hepatocellular carcinoma^8^ and aberrant FOXM1 expression contributes to cancer progression. Owing to the highly conserved sequences of the DNA‐binding Forkhead box and C‐terminal trans‐activation domains at the protein level, FOXM1 always exerts its function by directly targeting downstream genes.^27^ For example, FOXM1 regulates the cell cycle and cell proliferation by interacting with the promoter of kinesin family member (KIF) 4A.^8^ FOXM1 overexpression also affects epithelial‐mesenchymal transition (EMT) and tumorigenesis by activating the Snail promoter.^9^ Hence, targeting the downstream target genes of FOXM1 may provide a promising molecular therapy for various malignant tumours. However, the role of FOXM1 in tumorigenesis and progression, especially glycolysis, in NPC remains largely unknown. Interestingly, we found that FOXM1 was highly expressed in patients with NPC, and FOXM1 overexpression predicted a worse prognosis. The present study demonstrated that elevated expression of FOXM1 could positively regulate glycolysis by transactivating PDK1 promoter activity.

Aerobic glycolysis is preferentially used by cancer cells rather than oxidative phosphorylation, regardless of oxygen availability, despite being inefficient in generating ATP.^28^ Cancer cells adapting to glycolysis show increased glucose consumption, high lactate production, rapid ATP generation, and increased ECAR. Therefore, deciphering the mechanisms underlying aerobic glycolysis in cancer cells could lead to promising therapies for human malignancies. In this study, we observed that FOXM1 promoted aerobic glycolysis in NPC cells. As a transcription factor, FOXM1 has been shown to induce aerobic glycolysis in cancer cells to promote proliferation by directly regulating the transcriptional activation of multiple genes, including LDHA,^12^ GLUT1^29^ and HK2,^30^ suggesting that FOXM1 participates in metabolic reprogramming by targeting metabolic enzymes. Based on the above findings, and to clarify the molecular basis of FOXM1 in aerobic glycolysis, we investigated the key metabolic enzymes in NPC cells. Intriguingly, our results showed that the aberrant expression of FOXM1 had no significant effects on the mRNA levels of the indicated enzymes, except for PDK1, in NPC cells. Previous evidence has shown that PDKs (PDK1‐4), which are key glycolytic enzymes, are critical for regulating glucose metabolism. In recent years, targeting PDKs has gained increased attention in cancer therapy and disease management.^31^ PDK1 mRNA expression is elevated in human hepatocellular carcinoma, and Lin28 regulates PDK1 mainly via a post‐transcriptional mechanism, suggesting a novel rationale for targeting PDK1 for cancer therapy.^21^ In cervical cancer cells, N^6^‐methyladenosine positively regulates glycolysis via the induction of PDK4, providing new insights into the function of PDK4 in cancer therapy.^22^ Although the upregulation of PDKs is generally associated with the conventional Warburg effect, elevated FOXM1 expression is not closely associated with the mRNA levels of PDK2/3/4. The downregulation of PDK1 is correlated with a reduced phosphorylation of PDH, implying elevated enzyme activity.^32^ Interestingly, our results showed that the knockdown of FOXM1 significantly downregulated PDK1 expression, leading to reduced PDH phosphorylation. A previous study reported that a high expression of PDK1 leads to enhanced phosphorylation of the pyruvate dehydrogenase complex and reduced pyruvate decarboxylation into acetyl‐CoA.^32^ These results reveal a FOXM1‐induced metabolic switch that shunts glucose metabolites from the mitochondria to glycolysis, maintaining ATP production and supporting cell proliferation. Through in vitro and in vivo experiments, we proved that FOXM1 is involved in aerobic glycolysis by regulating PDK1 expression, most likely at the transcriptional level, in NPC cells.

Cancer progression is a complex process, and its cellular and molecular mechanisms remain to be determined. Accumulating data indicate that inhibiting glycolysis in cancer cells is a novel strategy for controlling cancer development.^7^ Our data demonstrated that FOXM1‐PDK1 signalling actively changed glucose metabolism from glucose oxidation to glycolysis, which promoted cancer development and progression in NPC. However, in mammals, PDK1 expression is strictly required from embryogenesis to adulthood.^19^ Systemic treatment with selective glycolysis inhibitors has adverse effects because glycolytic enzymes are essential for the biochemical function of normal cells. Therefore, targeting FOXM1 to inhibit glycolysis may provide a novel therapeutic approach for NPC treatment.

Considering the oncogenic role of FOXM1, we investigated the mechanism underlying FOXM1 regulation. It has been previously reported that the tumour microenvironment contributes to tumour progression and that cells exposed to severe hypoxia,^33^ reactive oxygen species^34^, ^35^ and cytokines^18^ in the tumour microenvironment are characterized by the stabilization and activation of HIF‐1α. Previous studies have shown that HIF‐1α transcriptionally regulates FOXM1 under hypoxic conditions.^35^ In the present study, we found that tumour microenvironment factors can induce the stabilization of HIF‐1α and increase the expression of FOXM1 in NPC cells under hypoxic and normoxic conditions. Activating the transcription of glycolytic genes by HIF‐1 α is required for metabolic changes to hypoxia through increased conversion of glucose to pyruvate and subsequently to lactate.^36^ Accumulating evidence revealed that the HIF‐1α‐mediated induction of PDK1 activates glycolysis.^37^, ^38^ However, the results in this study only evaluated the impact of FOXM1 on PDK1. The hypoxic environment‐induced FOXM1 regulation of PDK1, which further regulates glycolysis, was not explored in our results. Therefore, it is of great interest to further clarify the link between HIF‐1α, FOXM1 and glycolytic gene expression under normoxia or hypoxia.

In summary, our study is the first to provide compelling evidence demonstrating that FOXM1 can regulate glycolysis and the proliferation of NPC cells by regulating PDK1 expression. We identified a novel molecular mechanism of NPC glycolysis and progression and found that a new form of FOXM1‐PDK1 signalling is a promising molecular target for potential therapeutic strategies in NPC.

## AUTHOR CONTRIBUTIONS

**Qing Yang:** Data curation (equal); formal analysis (equal); investigation (equal); methodology (equal); software (equal); validation (equal); writing – original draft (equal); writing – review and editing (equal). **Fang Wu:** Project administration (equal); visualization (equal). **Yong Zhang:** Resources (equal); writing – review and editing (equal). **Rensheng Wang:** Conceptualization (equal); writing – review and editing (equal).

## CONFLICT OF INTEREST

The authors declare no conflicts of interest.

## Supporting information


Table S1‐S3

## Data Availability

The data supporting the findings of this study are available from the corresponding author upon reasonable request.
