# Supplementary material for: FOXM1 regulates glycolysis in nasopharyngeal carcinoma cells through PDK1
Source: J Cell Mol Med. 2022 Jun 3;26(13):3783–96. doi: 10.1111/jcmm.17413 (PMC9258706; doi:10.1111/jcmm.17413)
Supplement: Supplementary file 1 — Table S1‐S3 [file JCMM-26-3783-s001.docx]

| Supplementary Table 1. The primers for PCR | |
| --- | --- |
| Genes | Sequences |
| β-Actin | forward: 5’-CACCATTGGCAATGAGCGGTTC-3’  reverse: 5’-AGGTCTTTGCGGATGTCCACGT-3’ |
| FOXM1 | forward: 5’-GGCCATCCCCAACAATGCTA-3’  reverse: 5’-AGGTCTCCAGGGTCACTTCT-3’ |
| GLUT1 | Forward: 5’-AACCACTGCAACGGCTTAGA-3’  Reverse: 5’-TCACGGCTGGCACAAAACTA-3’ |
| HK2 | Forward: 5’-CCTGTGGCTTTFAAGACCT-3’  Reverse: 5’-CATGTTCACACACATCCGCC-3’ |
| LDHA | Forward: 5’-TTCAGCCCGATTCCGTTACC-3’  Reverse: 5’-CAAGGACCCACCCATGACAG-3’ |
| PDK1 | Forward: 5’-GCAAATCACCAGGACAGCC-3’  Reverse: 5’-ACCCAGCGTGACATGAACTT-3’ |
| PDK2 | Forward: 5’-TGCCTGTGAGAAAACCTCCT-3’  Reverse: 5’-TCTGGACATACCAGCTCTGC-3’ |
| PDK3 | Forward: 5’-CCCCTTTGGCTGGATTTGGT-3’  Reverse: 5’-AGGCGTGGTCTTGTAATGGC-3’ |
| PDK4 | Forward: 5’-GGTGGTGTTCCCCTGAGAAT-3’  Reverse: 5’-GCAAGCCGTAACCAAAACCA-3’ |
| PDK1 promoter  (-1771 to -1763) | Forward: 5’-ATTGTGCCCTGCCTAATCTGTT-3’  Reverse: 5’-CCTAACCAACTCCATCTTGCTTC-3’ |
| PDK1 promoter  (-1667 to -1661) | Forward: 5’-GAAGCAAGATGGAGTTGGTTAGG-3’  Reverse: 5’-AGGTGGAGCCTTTAGGAGAGAAT-3’ |

| Supplementary Table 2. Antibodies for western blot | | | |
| --- | --- | --- | --- |
| Antibodies | Source | Catalogue | Dilution |
| FOXM1 | CST | 20459 | 1:1000 |
| HK2 | CST | 2867 | 1:1000 |
| PDK1 | Abcam | ab202468 | 1:2000 |
| LDHA | Abcam | ab101562 | 1:1000 |
| GLUT1 | Abcam | ab115730 | 1:5000 |
| p-PDH 293 | Abcam | ab17746 | 1:1000 |
| HIF-1α | Proteintech | 20960-1-AP | 1:1000 |
| PDH-E1α | Proteintech | 66119-1-Ig | 1:5000 |
| α-Tubulin | Proteintech | 66031-1-Ig | 1:10000 |
| β-Actin | Proteintech | 66009-1-Ig | 1:5000 |
| anti-rabbit IgG | ZSGB-Bio | ZB-2301 | 1:5000 |
| anti-mouse IgG | ZSGB-Bio | ZB-2305 | 1:5000 |

| Supplementary Table 3. shRNA and siRNA target sequences | |
| --- | --- |
| Names | Sequence |
| shCtrl | 5’-TTCTCCGAACGTGTCACGT-3’ |
| shFOXM1#1 | 5’-GCTGGGATCAAGATTATTA-3’ |
| shFOXM1#2 | 5’-GCCAATCGTTCTCTGACAGAA-3’ |
| siCtrl | sense: 5’-UUCUCCGAACGUGUCACGUTT-3’  antisense: 5’-ACGUGACACGUUCGGAGAATT-3’ |
| siPDK1 | sense: 5’-GCCAAUACAAGUGGUUUAUTT-3’  antisense: 5’-AUAAACCACUUGUAUUGGCTT-3’ |
| siHIF-1α | sense: 5’-CUGAUGACCAGCAACUUGA-3’,  antisense: 5’-UCAAGUUGCUGGUCAUCAG-3’ |
